# Using GenAI for Socratic Questioning: An Approach to Higher‐Order Thinking for Nursing Education

**DOI:** 10.1002/nop2.70355

**Published:** 2025-10-24

**Authors:** Jackie Hoi Man Chan, Ken Hok Man Ho

**Affiliations:** ^1^ University of Wollongong in Dubai Dubai UAE; ^2^ School of Nursing and Midwifery La Trobe University Melbourne Victoria Australia

## 1 Introduction

Higher‐order thinking, such as critical thinking and reasoning, serves as both a prerequisite and an outcome of nursing competency, and is more important than ever in this AI era. Nurse educators and researchers, standing at the intersection of tradition, technology, and GenAI, seek to explore novel approaches that enable higher‐order thinking. Notably, around 60% of students globally engaged with ChatGPT in learning via direct questioning, attaining primarily lower‐order thinking (i.e., understanding and explaining), according to a recent global survey among higher education institutions (Ravšelj et al. 2025). Alarmingly, several recent reviews consistently suggested that employing GenAI as a content provider for quick answers encourages direct acceptance, which may contribute to limited critical thinking (Melisa et al. 2025; Raitskaya and Tikhonova 2025; Sardi et al. 2025). Even worse, potential metacognitive laziness overrelies on AI, offloads metacognitive load, and less effectively associates responsible metacognitive processes with learning tasks.

In the context of the nursing profession, philosophical thinking and person‐centred care extend beyond mere acceptance of knowledge. The nature of thinking about personhood and being empathetic requires continuous questioning, awareness, and reflection. To develop higher‐order thinking while using GenAI, the scoping reviews revealed a solution: embedding GenAI in pedagogical frameworks to provide reflective responses, guiding interpretation, justification, and reasoning—crucial elements of higher‐order thinking. Emerging evidence suggests pairing GenAI with Socratic questioning, an ancient pedagogy originating from the dialogues between the Greek philosopher Socrates and his students. This editorial serves to shed light on this novel learning approach in nursing education, discussing its relevance, rewards, and challenges.

## 2 An Ancient Pedagogy for Higher‐Order Thinking

Socratic questioning was first described in Plato's dialogues, illuminating the continuous questioning of concepts, assumptions, and beliefs (Overholser 1993). A famous quote from Socrates illuminates the philosophy of Socratic questioning.

An unexamined life is not worth living.

It highlights the importance of reflection, self‐awareness, and critical thinking, which were fostered among his students through the process of refuting and rebutting ideas. Socratic questioning facilitates learning through an iterative dialogue of thought‐provoking questioning and answering, probing the learners' reasoning and assumptions, and guiding them to formulate answers through logical reasoning and reflection, rather than providing them directly. It operates on three key dimensions: focused, exploratory, and spontaneous questioning. Focused questioning aims to initiate dialogue on a specific subject, inquiring about learners' assumptions and beliefs. Exploratory questioning aims to identify areas of clarity or confusion, uncovering inconsistencies and biases. Spontaneous questioning aims to maintain a fluid and dynamic dialogue, such as following an unanticipated line of thought. This deliberate and effortful thinking facilitates higher‐order thinking and internalisation of learning.

Socratic questioning is influential and has stood the test of time in nursing education and healthcare in modern days. Examples include Socratic‐style debriefing in simulation‐based learning and psychotherapy, particularly cognitive behavioural therapy. However, there are reported challenges, including training facilitators to ask open‐ended questions in a way that guides reflection and questioning of one's own assumptions, active listening, subject matter competence, managing large class sizes with diverse learning paces, and tolerance of uncertainty. Incorporating technology may overcome these human limitations and sustain the use of Socratic questioning in modern days.

## 3 Pairing GenAI With Socratic Questioning

Seizing the large language model, GenAI can simulate Socratic questioning by delivering a series of guiding questions that correspond to students' answers, just as a human teacher would. Therefore, students can move beyond passive reception of information, as in direct questioning, and are guided individually, thereby developing higher‐order thinking at their own pace. Indeed, this application is being piloted in universities as an AI agent to assist in student learning and the development of critical thinking. Institutions such as the University of Sydney impart Socratic questioning in AI wherein students' zone of proximal development is estimated based on their understanding of a particular topic. Then, probing questions are asked to guide the learning further, truly developing higher‐order thinking at a personal pace.

Notably, it has great potential in nursing education by gauging students' understanding of connections between concepts and translating knowledge into practice. To promote cognitive and affective learning, for example, we may prompt GenAI to simulate Socratic questioning to supplement teaching in nursing subjects, such as nursing leadership, ethics, and moral values. Learners interact with GenAI and are asked a series of questions about various styles of leadership and their applications in different situations, along with ethics and moral reasoning. This will guide learners to gain a deeper understanding and critical thinking, rather than relying solely on direct information from GenAI. Additionally, this may address the limitation of GenAI in providing non‐contextual specific information because it is not offered in Socratic questioning—only guiding questions are provided. To promote psychomotor learning, for example, we may employ GenAI alone or embed it in other technology, such as virtual reality simulations in learning wound dressing, where Socratic questioning guides learners' reasoning over the course of action. This approach fosters a deep understanding of each step in the procedure, rather than merely replicating the behaviours.

This pairing of ancient pedagogy and modern technology presents a novel and unique learning approach in advancing nursing education in the AI era. Strong leadership with effective strategies, including promoting GenAI literacy, institutional guidelines, enhancing prompting techniques, ensuring pedagogical alignment, and conducting pilot tests, is crucial for its integration. Failing to provide continuous support may result in ineffective delivery, potential frustration, and disengagement from learners and teachers. Therefore, promoting a culture of Socratic questioning via GenAI in learning and setting up the technology entails a structured implementation plan, which will be the next challenge for nursing educators. Meanwhile, the impact of GenAI‐based Socratic questioning on learning outcomes remains understudied. Current evidence is scant and non‐rigorous, limited to discussion papers, case studies, and quasi‐experimental studies. A great deal of evidence is necessary to provide a comprehensive picture. What are the students' and teachers' perceptions? What is the effect on critical thinking? What are the optimal dosages of direct and Socratic questioning via GenAI to achieve a synergistic effect? All these questions may inform the next research directions.

## 4 Summary

In sum, pairing GenAI with Socratic questioning holds immense potential in developing higher‐order thinking among nursing education. Instead of accepting direct information from GenAI, which is easy and efficient but lacks higher‐order thinking, formulating the answers through guided logical thinking is the way forward for nursing students. It is time to shift our application of GenAI from passive reception to active reasoning.

## Conflicts of Interest

The authors declare no conflicts of interest.

## Data Availability

Data sharing not applicable to this article as no datasets were generated or analyzed during the current study.
