# Correction to ‘Using GenAI for Socratic Questioning: An Approach to Higher‐Order Thinking for Nursing Education’

**DOI:** 10.1002/nop2.70467

**Published:** 2026-04-03

Man JH, Man KH. “Using GenAI for Socratic Questioning: An Approach to Higher‐Order Thinking for Nursing Education.” *Nursing Open*. 2025 12(10):e70355. https://doi.org/10.1002/nop2.70355.

The first and last names of authors are incorrect. The first name of first and second author should be “Jackie Hoi Man” and “Ken Hok Man”, respectively. In addition, the last name of first and second author should be “Chan” and “Ho”, respectively.

We apologise for this error.

